# Dead bird surveillance as an early warning system for West Nile virus.

**DOI:** 10.3201/eid0704.010405

**Published:** 2001

**Authors:** M Eidson, L Kramer, W Stone, Y Hagiwara, K Schmit, New York State West Nile Virus Avian Surveillance Team

**Affiliations:** Zoonoses Program, New York State Department of Health, Rm. 621 ESP Corning Tower, Albany, NY 12237, USA. mxe04@health.state.ny.us

## Abstract

As part of West Nile (WN) virus surveillance in New York State in 2000, 71,332 ill or dead birds were reported; 17,571 (24.6%) of these were American Crows. Of 3,976 dead birds tested, 1,263 (31.8%) were positive for WN virus. Viral activity was first confirmed in 60 of the state's 62 counties with WN virus-positive dead birds. Pathologic findings compatible with WN virus were seen in 1,576 birds (39.6% of those tested), of which 832 (52.8%) were positive for WN virus. Dead crow reports preceded confirmation of viral activity by several months, and WN virus-positive birds were found >3 months before the onset of human cases. Dead bird surveillance appears to be valuable for early detection of WN virus and for guiding public education and mosquito control efforts.


### Dead Bird Surveillance as an Early Warning System for West Nile Virus

**Millicent Eidson,* Laura Kramer,* Ward Stone,† Yoichiro Hagiwara,* Kate Schmit,* and The New York State West Nile Virus Avian Surveillance Team**[1]

*New York State Department of Health, Albany, New York, USA; and †New York State Department of Environmental Conservation, Delmar, New York, USA

As part of West Nile (WN) virus surveillance in New York State in 2000, 71,332 ill or dead birds were reported; 17,571 (24.6%) of these were American Crows. Of 3,976 dead birds tested, 1,263 (31.8%) were positive for WN virus. Viral activity was first confirmed in 60 of the state's 62 counties with WN virus-positive dead birds. Pathologic findings compatible with WN virus were seen in 1,576 birds (39.6% of those tested), of which 832 (52.8%) were positive for WN virus. Dead crow reports preceded confirmation of viral activity by several months, and WN virus-positive birds were found >3 months before the onset of human cases. Dead bird surveillance appears to be valuable for early detection of WN virus and for guiding public education and mosquito control efforts.

In the late summer and fall of 1999, New York State (NYS) had the first outbreak of West Nile (WN) virus encephalitis in the Western Hemisphere (1). The nucleotide sequence of the viruses isolated during this outbreak was most similar to that of a 1998 isolate from a goose in Israel (2). By the end of 1999, 62 human cases, 7 fatal, had occurred in New York City (NYC) and two neighboring counties, Nassau and Westchester (3).

Although WN virus infection was confirmed in dead birds shortly before it was confirmed in humans, no WN virus-positive dead birds were identified from time periods before the onset of symptoms in the first human cases, despite subsequent WN virus testing of birds collected earlier (4). Whether dead bird surveillance could provide an early warning for human WN virus cases could not be definitively established by analyses of 1999 data on dead bird surveillance. However, sightings of dead crows preceded laboratory confirmation of viral activity in any species, and testing of dead birds provided valuable information about the temporal and geographic spread of the virus (4).

We evaluate the usefulness of dead bird surveillance in 2000 for detecting geographic spread of WN virus and providing an early warning of the risk for transmission to humans. We also discuss lessons learned for other states that may be instituting a similar system.

### Methods

For WN virus surveillance, the New York State Department of Health (NYSDOH) developed and implemented an integrated electronic system based on the department's existing infrastructure for secure web-based electronic health information interchange with local health units, health-care facilities, and providers (5). The functional component of the infrastructure is called the Health Information Network, into which local health units entered data about sightings of ill or dead birds.

Freshly dead birds were submitted by local health units to the New York State Department of Environmental Conservation's Wildlife Pathology Unit for necropsy, which included evaluation of gross pathologic indications of WN virus infection and other possible causes of death. Organs collected for laboratory testing included brain, kidney, heart, liver, and spleen. Necropsy results were entered by the Wildlife Pathology Unit into the Health Information Network.

Local health units were permitted to send any species of birds for possible necropsy and WN virus testing. However, American Crows, Blue Jays, and Fish Crows, members of the Corvid family, which was most affected by the WN virus outbreak in 1999, were a top priority for submission, followed by raptors and house sparrows. As the outbreak progressed, birds from counties without documented WN virus were given higher priority, as well as migrating species of birds.

Most laboratory testing on dead birds was done at the NYSDOH Wadsworth Center, as described (6). WN virus infection was confirmed by at least two positive assays. Additional testing for overflow specimens was done at the National Wildlife Health Center laboratory in Madison, Wisconsin, as described (4).

Data from the Health Information Network were downloaded into Microsoft Excel and Microsoft Access files, and those software programs, along with SAS (Chapel Hill, NC), were used for descriptive statistical analyses. Microsoft PowerPoint was used for graphic representations of data and MapInfo (Troy, NY) for mapping. For data analysis, data were aggregated by report week, as requested by the Centers for Disease Control and Prevention for national surveillance.

Address for correspondence: Millicent Eidson, Zoonoses Program, New York State Department of Health, Rm. 621 ESP Corning Tower, Albany, NY 12237, USA; fax: 518-473-6590; e-mail: mxe04@health.state.ny.us

[1]Bryon Backenson, Kristen Bernard, Hwa-Gan Chang, Alan Dupuis, Gregory Ebel, Ivan Gotham, Susan Jones, Elizabeth Kauffman, Dale Morse, John Napoli, Perry Smith, Charles Trimarchi, Barbara Wallace, Dennis White, and Amy Willsey, New York State Department of Health.

### Results

For 2000, 71,332 ill or dead birds, of which 17,571 (24.6%) were American Crows, were reported through the Health Information Network. Of 3,976 dead birds tested by NYSDOH's Wadsworth Center or the National Wildlife Health Center, 1,263 (31.8%) were positive for WN virus. These WN virus-positive birds represented 63 species, 30 families, and 14 orders (7); most were American Crows (846 birds, 67%).

Most of the ill or dead birds (62,339 [87.4%]) were found singly. For sightings of multiple birds, the number of birds reported ranged from 2 to 100 (mean 2.8). Only 675 (0.95%) of the birds were seen alive and ill; the others were reported as dead. Symptom information was provided for 582 of the ill birds, with "neurologic signs" listed for 413 (71%). Four of these tested positive for WN virus after death.

Of the dead birds tested for WN virus, 1,576 (39.6%) had one or more signs compatible with WN virus (8), such as emaciation, splenomegaly, hepatomegaly, cardiac or pericardial lesions, or possible signs of encephalitis (Table). Of these birds, 832 (52.8%) subsequently tested positive for WN virus (overall positive predictive value for pathologic findings). Before the onset date for the first human case in NYS in 2000 (July 20), the sensitivity of gross pathologic findings (the proportion of WN virus-positive birds that had suspicious pathology) was highest in American Crows (51.8%). The overall positive predictive value (PPV) for pathologic findings was 27.9% for this time period. The overall specificity for the necropsy evaluation was high for most species tested, with 90.3% of WN virus-negative birds having no gross pathologic indication of WN virus. The negative predictive value (NPV) for necropsy evaluation was 85.3% before the onset of human cases.

For birds collected on or after the human case onset, the overall sensitivity and PPV increased to 68% and 55.1%, respectively, while the specificity and NPV decreased to 62.1% and 73.9%, respectively. The least sensitive species was the House Sparrow; 18.8% of those testing positive had pathologic signs on necropsy. Before the onset of the first human case, American Crows had significantly higher sensitivity, PPV, specificity, and NPV than other species combined. After the onset of the first human case, crows were significantly higher in sensitivity and PPV but significantly lower in specificity and NPV ($p<0.05$). When values for all species combined before human case onset were compared with values after onset, sensitivity and PPV significantly increased, while specificity and NPV significantly decreased ($p<0.001$).

Signs of trauma were found on necropsy in 1,885 (47%) of the birds tested for WN virus. Of these birds, 480 (25.5%) subsequently tested positive for WN virus (PPV). In comparison, 1,308 (63%) of the 2,091 birds without trauma tested negative for WN virus (NPV). American crows without trauma were significantly more likely to test positive for WN virus (568 [49.1%] of 1,158) than crows with trauma (278 [32.9%] of 845) ($p<0.001$).

The first laboratory confirmations that the virus was still present in the United States were from areas affected in 1999: isolations in February 2000 of virus from a mosquito pool in New York City (9) and a hawk in Westchester County (tested by the University of Connecticut and the Connecticut Agriculture Experiment Station) (Figure 1, bars). However, the first evidence of viral transmission during the 2000 season was two dead crows collected in Rockland County (a county in the lower Hudson Valley affected by the outbreak in 1999) on May 22 and confirmed as positive for WN virus on June 9. One crow from Suffolk County, Long Island (another area affected by the outbreak in 1999), found dead on April 1, 2000, frozen until August, then submitted for laboratory testing, also was confirmed as positive for WN virus, making it the earliest identification of viral activity in the 2000 mosquito season.

Table. Sensitivity, specificity, positive predictive value (PPV), and negative predictive value (NPV) of pathology results[a] for West Nile (WN) virus, New York State, 2000, before and after onset of first human case on July 20

| | No. pos. on WN virus testing | | No. neg. on WN virus testing | |
|---|---|---|---|---|
| Species | No. pos. on necropsy (%)[b] | No. neg. on necropsy | No. pos. on necropsy | No. neg. on necropsy (%)[c] |
| **Jan 1 - Jul 19** | | | | |
| American Crow[d] | 29 (51.8) | 27 | 34 | 551 (94.2) |
| Blue Jay | 7 (25) | 21 | 37 | 101 (73.2) |
| Fish Crow | 0 (--) | 2 | 1 | 18 (94.7) |
| American Robin | 0 (--) | 0 | 5 | 18 (78.3) |
| House Sparrow | 0 (--) | 1 | 2 | 35 (94.6) |
| Other species | 2 (25) | 6 | 19 | 186 (90.7) |
| Total[e] | 38 (40) (PPV=27.9%) | 57 | 98 | 909 (90.3) (NPV=85.3%) |
| **Jul 20 - Dec 31** | | | | |
| American Crow[d] | 624 (79.0) | 166 | 303 | 269 (47.0) |
| Blue Jay | 76 (61.3) | 48 | 124 | 126 (50.4) |
| Fish Crow | 16 (84.2) | 3 | 10 | 3 (23.1) |
| American Robin | 7 (43.8) | 9 | 32 | 16 (33.3) |
| House Sparrow | 3 (18.8) | 13 | 11 | 32 (74.4) |
| Other species | 68 (33.5) | 135 | 166 | 614 (78.7) |
| Total[e] | 794 (68.0) (PPV=55.1%) | 374 | 646 | 1,060 (62.1) (NPV=73.9%) |
| **Total (all year)** | 832 (65.8%) | 431 | 744 | 1,969 (72.6%) |

[a]Gross postmortem signs considered indicative of possible WN virus infection included one or more of the following: emaciation, splenomegaly, hepatomegaly, cardiac or pericardial lesions, and possible signs of encephalitis.
[b]Sensitivity of pathologic findings on gross necropsy for detecting WN virus.
[c]Specificity of pathologic findings on gross necropsy for ruling out WN virus.
[d]Differences between American Crows and other species combined significant at 0.05 level.
[e]Differences between time periods (all species combined) significant at 0.001 level.

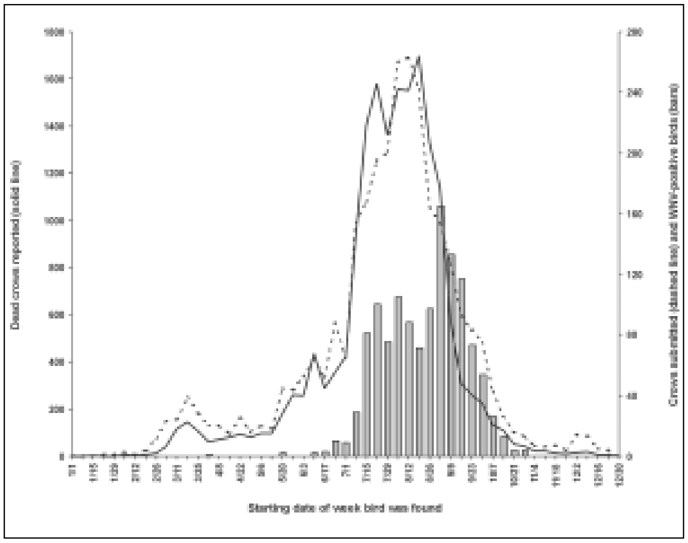

Figure 1. Sightings of ill or dead crows, dead crows submitted for possible West Nile virus testing, and West Nile virus-positive dead birds (all species) by week, New York State, 2000.

The numbers of ill or dead crow reports remained low (<10 per week) early in the year (Figure 1, solid line). Increases in dead crow sightings occurred just before the collection date for the first WN virus-positive crow of the season on April 1 and the same week that the first crows to be identified as positive were found in May, even though the results were not known until 2 weeks later. The steep increase in dead crow sightings in early July predates the onset date for the first human case (July 20) and the increase in WN virus-positive birds by several weeks. Although only a small proportion of the ill or dead crows seen were submitted for possible necropsy and WN-virus testing (Figure 1, dashed line), the number of crows submitted closely parallels the number of crows seen and reported over time.

With regard to geographic spread of the virus, dead crow reports during January-March were concentrated in the areas affected by the outbreak in 1999, as well as into the Hudson River Valley. During the period before the onset of the first human case (Figure 2a), dead crow reports increased to 4,600 in these areas, and sightings began to occur along other bodies of water, including Lake Champlain in the northeastern corner of the state, the Mohawk River and various lakes in central NYS, and Lake Erie and Lake Ontario in western NYS. Many of the state's largest cities (by human population size) are also in some of these same areas. In the period after human WN virus cases began to occur (Figure 2b), 12,530 dead crows were sighted; the highest number were from counties with viral activity in 1999. Increased expansion of reports into other counties of the state clustered around bodies of water and some population centers.

The geographic spread of the virus, as indicated by surveillance with laboratory testing of dead birds, was similar but lagged behind the dead crow reports by several months. Before the first human case, the 91 WN virus-positive dead birds in 2000 were confined primarily to the four counties near NYC with viral activity in 1999 and two of NYC's five boroughs, although WN virus-positive birds were also found in four upstate counties (Figure 2c). Subsequently, 1,171 WN virus-positive birds were reported from all but one NYS county and all five NYC boroughs (Figure 2d). The first WN virus-positive bird found in 2000 outside the 1999 outbreak area was a Rock Dove collected in central NYS on July 6.

For the first laboratory confirmation of viral activity in 2000 in 60 of the 62 NYS counties and NYC boroughs, 30 (50%) had an American Crow, 8 (13.3%) had a Blue Jay, 1 had a Fish Crow, and 21 (35%) had other bird species. One county reported a positive mosquito pool before a positive bird. The first positive "other" species included House Sparrow, Song Sparrow, Ovenbird, Catbird, Robin, Cedar Waxwing, Ruffed Grouse, Rock Dove, Mourning Dove, European Starling, Wood Thrush, Common Grackle, Ring-billed Gull, Greater Black-backed Gull, Mute Swan, Great Horned Owl, Cooper's Hawk, American Kestrel, and Red-tailed Hawk. For the counties without an American Crow or other corvid as their first positive bird species, confirmation of viral activity would have been delayed 1 to 47 days (median 13) or 1 to 41 days (median 11), respectively, if noncorvid species had not been tested. Fifteen counties with viral activity confirmed by dead bird testing (25%) never had a WN virus-positive American Crow, and nine counties never had a WN virus-positive corvid.

### Conclusion

At the end of 1999, it was unknown whether a human outbreak of WN virus would recur and whether dead bird surveillance could detect any reappearance of viral activity before human infection. A dead bird surveillance system (established in NYS in 1999 after the bird and human WN virus outbreaks were recognized) was refined for 2000 to include real-time reporting of dead bird sightings by all local health units, using the state's web-based Health Information Network and laboratory testing by the NYSDOH's Wadsworth Center. In 2000, dead bird surveillance (both dead crow sightings and laboratory testing of birds) provided an early warning of WN virus activity before the first human case in NYS, both temporally and geographically. However, test results for many of the WN virus-positive birds were not known soon enough to guide prevention and control activities before the onset of illness in the first human case.

The earliest warning was provided by the dead crow sightings, with the geographic distribution of dead crow reports from earlier time periods overlapping that of WN virus-positive birds from later time periods. Before the first human case, the wider distribution of dead crow sightings compared with the distribution of WN virus-positive birds may reflect the amount of testing done. Although submissions of crows for testing occurred in proportion to the level of dead crow sightings (Figure 1), the number of birds submitted for testing may have been insufficient to confirm low levels of viral activity in some areas.

To provide an early warning of viral activity, dead bird surveillance requires capacity at the local level to let the public know where to report dead birds, as well as a system for answering phone calls, recording data, and collecting birds for testing. Resources for bird necropsies and laboratory testing are also required. The usefulness of this system for monitoring WN virus is influenced by the amount of effort expended by the public and local agencies to notice and report the dead birds. Unlike ill humans, ill or dead birds are dependent on humans to observe and investigate their condition.

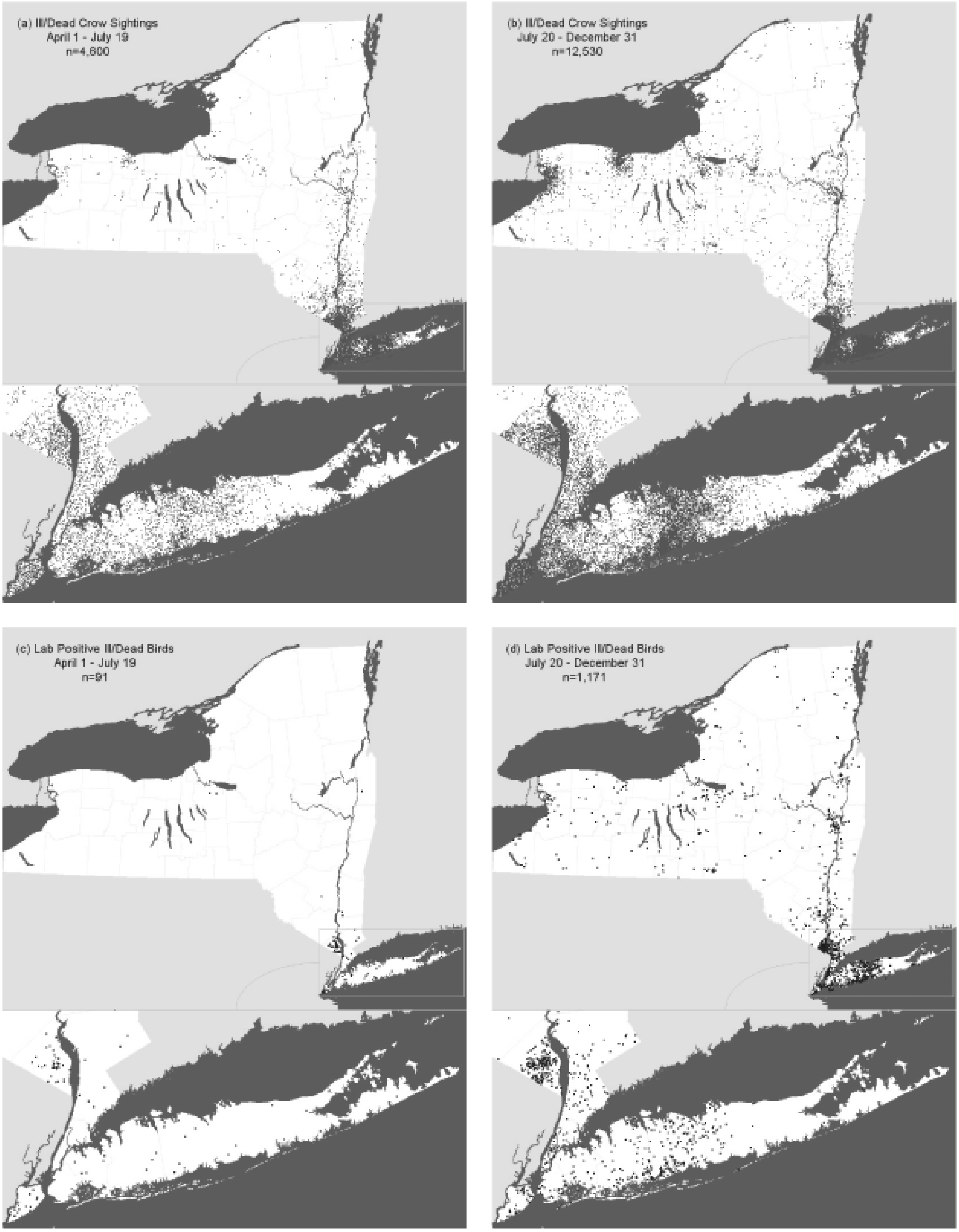

Figure 2. Maps of ill or dead crow sightings (a,b) and West Nile virus-positive dead birds of any species (c,d), New York State, 2000.

Successful dead bird surveillance can be based on a number of factors, including frequency and extent of information provided to the public to encourage reporting of dead birds, the number of people living in an area to see dead birds, and enhanced public interest when new WN virus findings or reports are issued. Potential limitations to dead bird surveillance for WN virus include absence of or scarcity of American Crows in some geographic areas or the possibility that crows will become increasingly immune to WN virus, with a consequent reduction in their case-fatality rate.

Because of the resources required for reporting and testing dead birds, agencies responding to WN virus must make decisions about whether to cast a wider net, with a more sensitive surveillance system capable of detecting the earliest viral activity, or a narrower net, with a more specific surveillance system that eliminates birds less likely to have WN virus. To provide the earliest warning of viral activity to encourage subsequent surveillance, prevention, and control, we recommend unrestricted testing by species, presence of trauma, number of dead birds seen in the area, or pathologic findings before laboratory confirmation of viral activity in an area. Once viral activity has been confirmed, laboratory testing may be conducted primarily to verify continued viral activity, and more specific submission criteria, such as restrictions to American Crows without trauma and with compatible pathologic findings, may be adopted to conserve scarce laboratory resources.

### Acknowledgments

The authors thank the following for their assistance in avian surveillance: the health directors and staff of the New York City Department of Health and the New York State county health departments; Joan Cleary-Miron, Doug Docherty, Linda Glaser, Wallace Hansen, Robert McLean, Bill Moyer, Kiet Ngo, Joseph Okoniewski, Michelle Przedwiecki, John Rososki, Robert Rudd, Morris Safford, Jr., Pei Yong Shi, Art Sulgher, Deb Sottolano, and Joseph Therrien.

We acknowledge CDC grants U66CCU21531203 (Demonstration Projects to Promote Integrated Public Health Information Systems) and U90CCU21698801 (Bioterrorism and Health Alert Network/Training grant), and CDC Cooperative Agreement U50CCU212415 (Epidemiology and Laboratory Capacity for Infectious Disease) for their contributions to surveillance infrastructure, analyses, and laboratory capacity.

Dr. Eidson is State Public Health Veterinarian and Director of the Zoonoses Program, New York State Department of Health. In addition, she is an associate professor in the Department of Epidemiology, State University of New York School of Public Health, and a diplomate of the American College of Veterinary Preventive Medicine. Her research focuses on rabies and West Nile virus.

### References

1. Centers for Disease Control and Prevention. Update: West Nile Virus encephalitis—New York, 1999. MMWR Morb Mortal Wkly Rep 1999;48:944-6,955.
2. Lanciotti RS, Roehrig JT, Deubel V, Smith J, Parker M, Steele K, et al. Origin of the West Nile virus responsible for an outbreak of encephalitis in the northeastern United States. Science 1999;286:2333-7.
3. Nash D, Mostashari F, Fine A, Miller J, O'Leary D, Murray K, et al. Outbreak of West Nile virus infection, New York City area, 1999. N Engl J Med 2001. In press.
4. Eidson M, Komar N, Sorhage F, Nelson R, Talbot T, Mostashari F, et al. Crow Deaths as a Sentinel Surveillance System for West Nile Virus in the Northeastern United States, 1999. Emerg Infect Dis 2001;7:615-20.
5. Gotham IJ, Eidson M, White DJ, Wallace BJ, Chang HG, Johnson GS, et al. West Nile virus: A case study in how New York State health information infrastructure facilitates preparation and response to disease outbreaks. Journal of Public Health Management and Practice 2001;7(5):79-89.
6. Shi P-Y, Kauffman EB, Ren P, Felton A, Tai JH, Dupuis II AP, et al. High throughput detection of West Nile virus RNA. J Clin Microbiol 2001;39:1264-71.
7. Bernard KA, Maffei JG, Jones SA, Kauffman EB, Ebel GD, Dupuis AP, et al. West Nile virus infection in birds and mosquitoes, New York State, 2000. Emerg Infect Dis 2001;7:679-85.
8. Steele KE, Linn MJ, Schoepp RJ, Komar N, Geisbert TW, Manduca RM, et al. Pathology of fatal West Nile virus infections in native and exotic birds during the 1999 outbreak in New York City, New York. Vet Pathol 2000;37:208-24.
9. Centers for Disease Control and Prevention. Update: Surveillance for West Nile virus in overwintering mosquitoes—New York, 2000. MMWR Morb Mortal Wkly Rep 2000;49:178-9.
